# The Impact of TPA Auxiliary Donor and the π-Linkers on the Performance of Newly Designed Dye-Sensitized Solar Cells: Computational Investigation

**DOI:** 10.3390/ma16041611

**Published:** 2023-02-15

**Authors:** Si Mohamed Bouzzine, Alioui Abdelaaziz, Mohamed Hamidi, Fatimah A. M. Al-Zahrani, Mohie E. M. Zayed, Reda M. El-Shishtawy

**Affiliations:** 1 Regional Center for Education and Training Professional, B.P. 8 Errachidia, Morocco; 2 Equipe de Chimie-Physique, Electrochimie et Environnement, Laboratoire de Chimie-Physique, Environnement et Matériaux, Université Moulay Ismaïl, B.P. 509 Boutalamine, Errachidia, Morocco; 3 Chemistry Department, Faculty of Science, King Khalid University, P.O. Box 9004, Abha 61413, Saudi Arabia; 4 Chemistry Department, Faculty of Science, King Abdulaziz University, Jeddah 21589, Saudi Arabia; 5 Dyeing, Printing and Textile Auxiliaries Department, Textile Research and Technology Institute, National Research Centre, 33 EL Buhouth St., Dokki, Giza 12622, Egypt

**Keywords:** dye-sensitized solar cells, di-anchoring, TD/TD-DFT, D_1_-D_2_-π-linker-π-(A)_2_, photovoltaic properties

## Abstract

The efficiency of the newly designed dye-sensitized solar cells (DSSCs) containing triphenylamine, diphenylamine (TPA), phenothiazine, and phenoxazine as donors and triazine, phenyl with D_1_-D_2_-π-linker-π-(A)_2_ architecture has been investigated using density functional theory (DFT) and time-dependent (TD-DFT) methods. These methods were used to investigate the geometrical structures, electronic properties, absorption, photovoltaic properties, and chemical reactivity. Furthermore, the calculated results indicate that different architectures can modify the energy levels of HOMO and LUMO and reduce the energy gap. The absorption undergoes a redshift displacement. This work aims at calculating the structural geometries and the electronic and optical properties of the designed dyes. Furthermore, the dye adsorption characteristics, such as the optoelectronic properties and the adsorption energies in the TiO_2_ clusters, were calculated with counterpoise correction and discussed.

## 1. Introduction

An increase in the world population and the increasing consumption of fossils and the more severe environmental pollution crisis contribute immensely to high-energy demand. In addition, growing world energy demand and limited oil and coal reserves will limit future economic development. It is, therefore, necessary to exploit renewable energy sources, such as solar energy, to maintain sustainable social and economic development. Solar energy is widely recognized as the most promising candidate for helping solve this problem. In this interest, the search for an efficient method for harnessing solar light conversion to electricity using dyes-sensitized solar cells (DSSCs) has been investigated [1,2,3,4,5,6,7]. The solution-processable photovoltaic DSSCs have the advantages of being clean, cheap, renewable, inexhaustible, pollution-free, and large-scale production [8,9,10,11,12,13,14]. Because of their lower production cost, DSSCs offer a viable alternative to conventional all-inorganic solar cells. Over the past decades, DSSCs have attracted a great deal of attention as an alternative to silicon solar cells because they use environmentally friendly materials through inexpensive processes and offer commercially feasible energy conversion efficiency [15,16,17].

Numerous attempts at molecular modification based on dyes characteristic of D-π-A have been carried out to improve the photoelectric performance of DSSC devices. There are many new dyes with new designs, such as D-π-A, whose theoretical study was interested in the performance of photovoltaic properties [18,19,20]. Sometimes we find dyes where we have double donor D-D-π-A or double acceptor D-A-π-A or double π-spacer D-π-A-π-D [21,22,23,24]. S. Gauthier et al. synthesized di-anchoring type D-(π-A)_2_ dyes with a PEC of 5.23% [25], whereas M. B. Desta et al. studied three new D-(π-A)_2_ di-anchoring organic dyes comprising an arylamine as the electron donor with a maximum of PEC 6.69% [26]. Other types of dyes, L(D-π-A)_2_ di-anchoring organic, were investigated by Z. Wang et al. [27].

This new dye design, which could enhance the D-π-A concept, emphasized the possibility that ordinary organic dyes’ single anchor group would be a drawback in comparison to dyes containing up to four anchor groups [28,29,30]. As a result, the multi-anchoring dye concept was put forth for the conventional D-π-A architectural design. The interest in this design strategy is justified for a number of reasons. The two bridges in the more complex system should primarily result in an expansion of the absorption at longer wavelengths, a broader absorption profile, and an increased molar extinction coefficient when compared to those of D-A, all of which improve light harvesting. Another electron withdrawing unit (A) might also help improve the organic dye’s photostability by lowering its HOMO-LUMO divergence [31].

**Figure 1:**
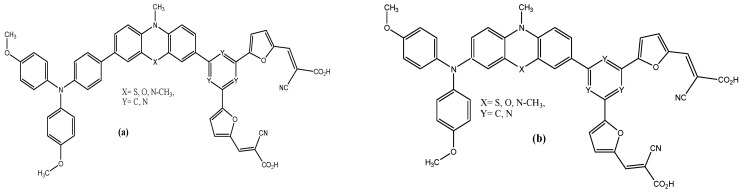
Chemical structures of the designed D_1_-D_2_-π-linker-π-(A)_2_ sensitizers (**a**) with triphenylamine (**b**) with diphenylamine.

The present contribution aims to explore better the D_1_-D_2_-π-linker-π-(A)_2_ structures (Figure 1) by studying the effects of the change of donor (triphenylamine or diphenylamine) and (phenothiazine or phenoxazine), as well as the impact of pi-linkers (phenyl or triazine) on the geometric and optoelectronic properties of the studied dyes that contain the cyanoacrylic acid group as acceptors, via furan spacers, using density functional theory (DFT) and time-dependent DFT (TD-DFT).

## 2. Computational Methods

All calculations were carried out using the Gaussian 09 program package [32]. According to our previous study [33,34], in which the efficiency and robustness of various hybrid and meta-hybrid functional, such as B3LYP [35,36] and BHandH [37], are used in conjunction with their basis-set 6-31G(d,p) for non-metal atoms and LANL2DZ for Ti atoms [38,39,40] for simulating the geometrical and electronic properties and absorption spectra. The BHandH [37] optimizes the ground state geometry without any symmetry constraint, while the functional BHandH (which includes a fraction of 50% HF exchange) is used to record the UV-vis absorption spectra using a Poples large basis-set 6-31G(d,p) for the soft atoms (H, C, N, O, S) and effective core potential LANL2DZ basis set for titanium atoms [41]. The geometrical optimizations and the absorption spectrum simulations were performed in the chloroform medium using the implicit CPCM [42] model (conductor-like polarizable continuum model). The complexation energy of the Dye@TiO_2_ clusters is calculated with the corrected counterpoise method [43], taking into account the Basis set superposition errors (BSSEs) [44,45].

## 3. Results and Discussion

### 3.1. Geometrical Properties

**Figure 2:**
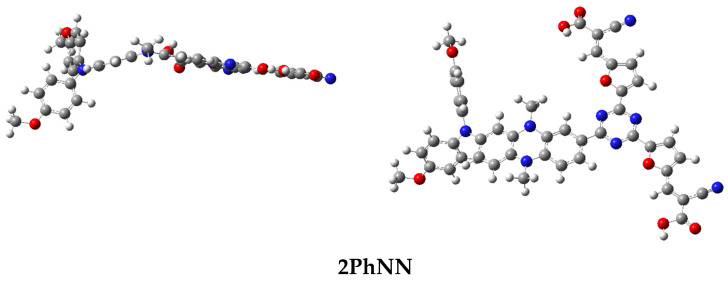

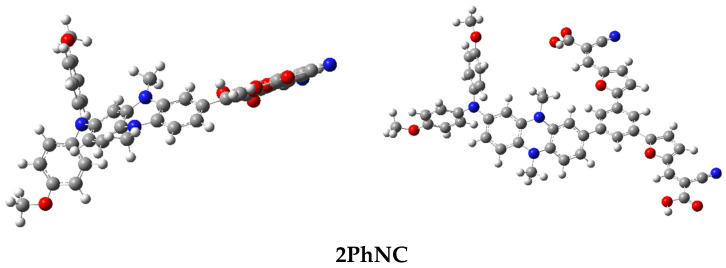
Optimized geometry of 2PhNN and 2PhNC (the other structure is inserted in Figure S1).

As we know, the efficient process of electron transfer and the electronic and optical properties are better with the coplanarity of the geometrical structure of colorants [46]. The structure optimizations of the studied dyes were performed using the B3LYP/6-31G(d, p) method [36], and the selected geometrical parameters, bond lengths, and dihedral angles are listed in Figure 2 and Table 1. According to the obtained results (Table 1), we can deduce that the θ values, which are the dihedral angles between the acceptor unit (A) and the π-bridge unit (π), are in order 37, 57°, and 35,4°, respectively, for dyes based triphenylamine and diphenylamine using phenyl as a bridge. The observed non-planar structures for all dyes are likely the result of steric effects between the hydrogen of phenyl (π-spacer) and phenothiazine or phenoxazine of the adjacent group, which would contribute to the suppression of dye aggregation problems and charge recombination [47,48]. On the other hand, the dihedral angles θ of dyes bridged by triazine are coplanar. Therefore, the nature of the bridging group has a minor impact on the π-bridge fragment flatness, which will facilitate electron delocalization and thus improve the intramolecular charge transfer and photovoltaic properties of the DSSC [49]. In addition, the values of the distances $d_{i}$(i = 1 − 2) of all dyes are in the range of 1.410−1.481 Å. These values are lower than the C-C single bonds (~1.530 Å) [49], which confirms the strong resonance between the donor and acceptor for all dyes.

### 3.2. Electronic Properties

Using B3LYP/6-31G, the energy gap E_gap_ for the study dyes was calculated from the discrepancies in HOMO and LUMO energy levels (d,p). Table 2a,b contain the results without any restrictions, and the optimization was carried out in the gas phase. The calculated values of orbital HOMO in the studied dyes are between −4.61 eV to −4.88 eV for the first series and between −4.64 eV to −4.97 eV for the second series (Table 2a,b). The energy of the LUMO orbitals has an average of about −3.20 eV for the phenyl-mediated compounds. Whereas, for triphenyl and diphenyl dyes, compounds containing triazine rings show a value of −3.33 eV and −3.42 eV, respectively. The previous results show that the gap value decreases for the same dyes upon replacing phenyl rings with triazine rings. This can only be due to the effect of substituting carbon with nitrogen (phenyl to triazine).

**Figure 3:**
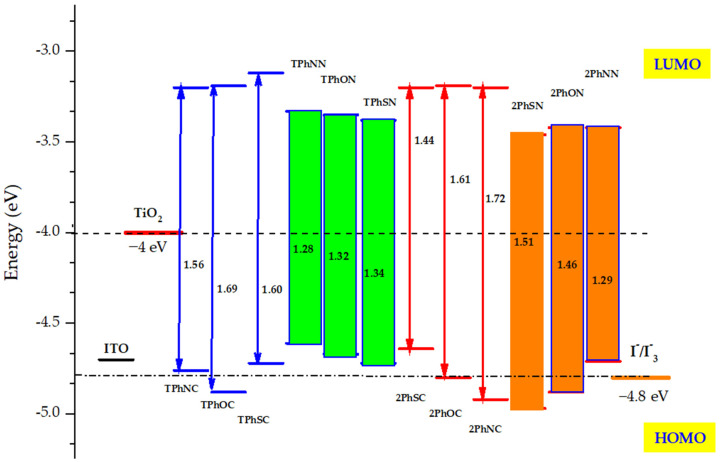
Frontier molecular orbital energy levels and HOMO-LUMO bandgap of all isolated dyes calculated at B3LYP/6-31G (d, p) level, along with the energy of TiO_2_ conduction band and the redox potential of $I^{-}/I_{3}^{-}\;$electrolyte.

Differently, TPhOC, 2PhNC, 2PhSN, and 2PhON chromophores’ estimated level of HOMO energies are more negative than those of other dyes, while their LUMO values are more positive than those of the TiO_2_ conduction band (Figure 3). On the other hand, the HOMO values of these dyes are lower than the $E(I^{-}/I_{3}^{-})$ redox potential. These HOMO positions indicate that the oxidized form the reduced species in the electrolyte to investigate efficient charge separation. Furthermore, this regeneration was affected by the nature of the donor and the π-spacers block. This result indicates that these dyes can regenerate the charge and thus can be used in dye-sensitized solar cells.

**Figure 4:**
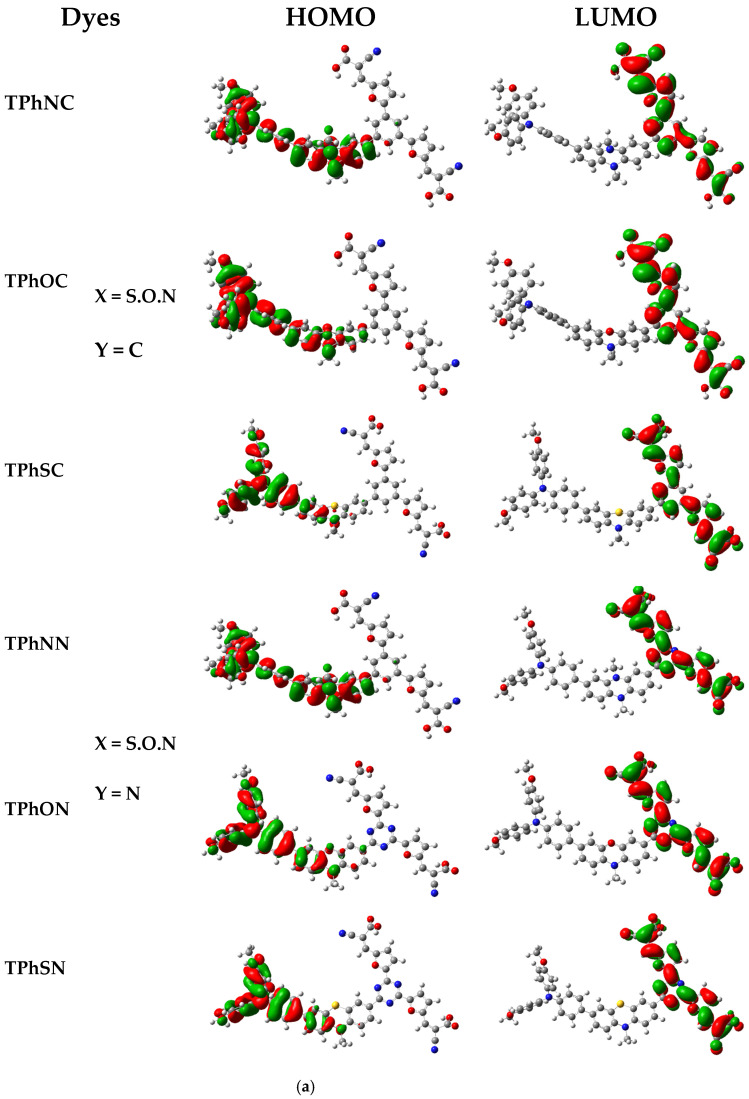

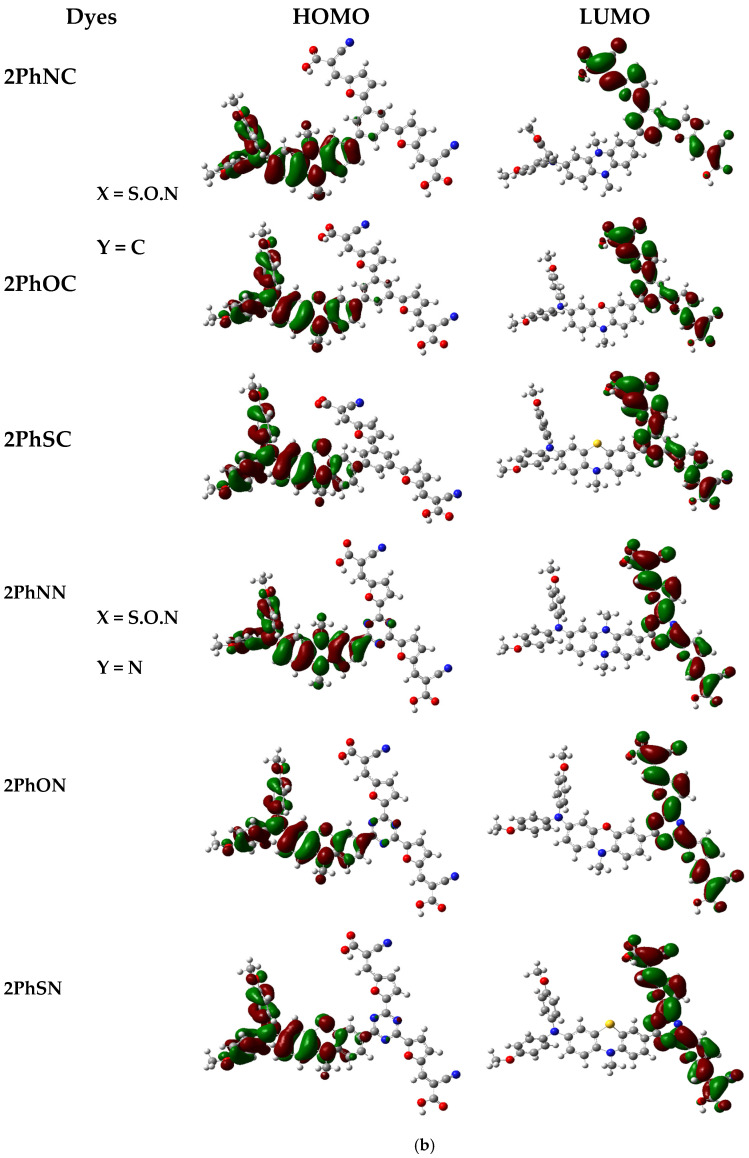
(**a**) Frontier Molecular Orbitals (FMOs) of dyes-based triphenyl amine (TPh) using B3LYP/6-31G(d,p). (**b**): Frontier Molecular Oritals of dyes-based diphenyl amine (2Ph) using B3LYP/6-31G(d,p).

**Figure 5:**
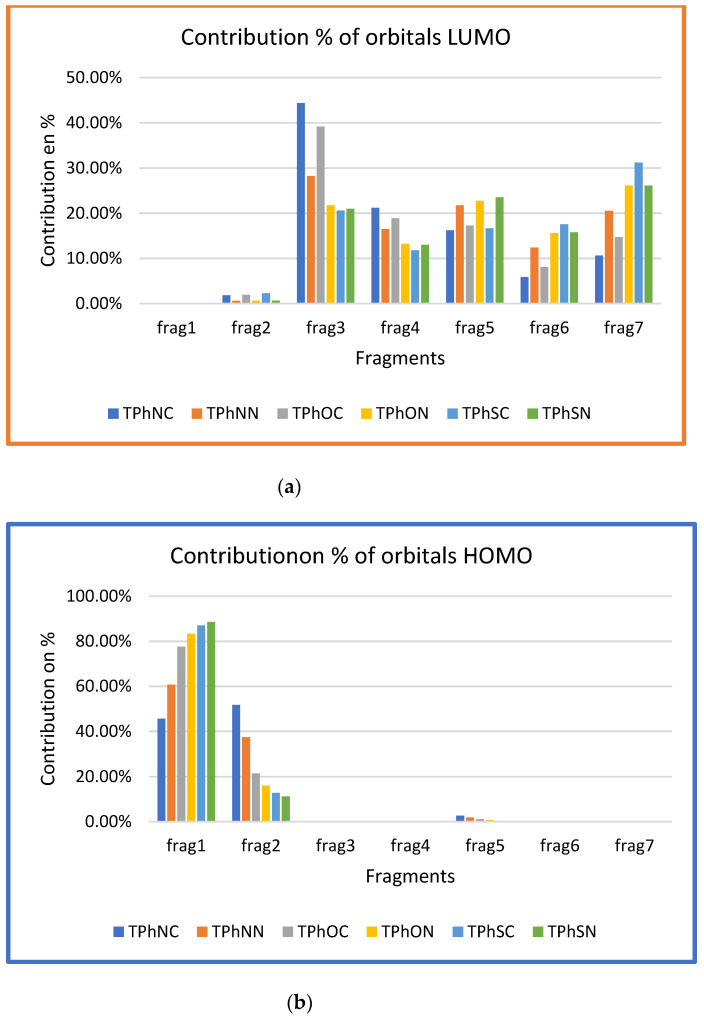
(**a**) Contribution of molecular LUMO orbitals of different fragments for the studied dyes. (**b**) Contribution of molecular HOMO orbitals of different fragments for the studied dyes.

Figure 4a,b, in which the electronic density of the HOMO of all compounds is primarily distributed on the donor (D1 = frag1 and D2 = frag2; see Figure S2). Figure 4a, with high electron density, shows the distribution of the electronic density, as indicated by the HOMO and LUMO orbitals, respectively, which contributed to the electron transition. While in the π-linker and the acceptor (π-linker-π-(A)_2_), there is a lack of density in the neighboring rings, which is checked by calculating the density contribution (Figure 5a,b). In contrast, the electronic density of the LUMO orbital is mainly displaced at the acceptor and the π-spacer segment (frag3-frag4-frag5-frag6-frag7) (Figure S2). This explains very well the transfer of load from the donor to the acceptor through the spacer.

### 3.3. Photovoltaic Properties

#### 3.3.1. Driving Force

The theoretical background for the photovoltaic parameters is provided in the supplementary file. The $\Delta G^{inject}$ are negative values for the studied dyes, and the absolute values of ($\Delta G^{inject}$) are above 0.2 eV. This indicates that the process is spontaneous and favors the introduction of electrons into the conduction band of the semiconductor TiO_2_ during stimulation of the dye’s excited state [50,51,52]. Table 3a shows that the injection drive $\Delta G^{inject}$ of the dyes decreases in the order of TPhNN > TPhON > TPhSN > TPhNC > TPhOC > TPhSC for the triphenyl-based donor dyes, but when the donor is biphenyl amine, the injection drive $\Delta G^{inject}$ of the dyes decreases in the order of 2PhNN > 2PhNC > 2PhSN > 2PhOC > 2PhOC > 2PhSC (Table 3b).

Implying that TPhSC, TPhOC, and 2PhOC, the absolute values of ($\left|\Delta G^{inject}\right|$) than other dyes, which conduced to faster injection. Consequently, such dyes possess large $J_{SC}$ compared to the other examined. The process of unfavorable charge analyzation is also analyzed. The greater the $\Delta G^{rec}$ value, the easier the charge recombination [53], and enhancing charge separation and reducing charge recombination are efficient ways to raise photoelectric conversion efficiency [54]. The $\Delta G^{rec}$ values are negative for all the dyes, indicating that these processes are thermodynamically favorable. The average value of G for the dyes based on triphenylamine is of the order of −0.72 eV. In contrast, this value is of the order of −0.82 eV for the series whose donor is the diphenylamine, which shows that the second series has a high chance of charge recombination.

Electron transfer from the redox electrolyte is required to renew the dye, which is then reduced at the counter electrode. The calculated values of $\Delta G^{reg}$ are listed in Table 3a,b. They show positive values except for the TPhOC, 2PhNC, 2PhON, and 2PhSN, suggesting these dyes must be regenerated by electron transfer from the redox electrolyte and then reduced at the counter electrode. This regeneration was in accordance with the low HOMO level of 2PhSN followed by 2PhNC, 2PhON, and TPhOC relative to the redox electrolyte potential.

#### 3.3.2. Absorption Spectra

**Figure 6:**
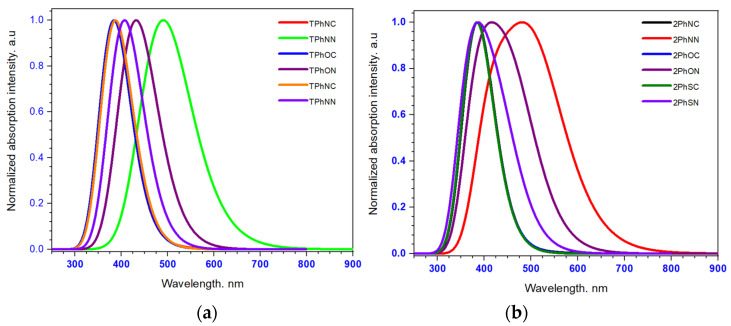
The absorption spectra of dye-based triphenylamine (**a**) diphenylamine (**b**).

The conductor-like polarizable continuum model (C-PCM) level of theory functional associated with the excitation was used to determine the absorption spectra using excitation based on the optimization ground-state geometry in a CH_2_Cl_2_ solvent by the TD-BHandH/6-31G(d,p) level of theory functional. The pertinent photophysical indexes of twelve dyes are displayed in Figure 6, and their corresponding results are summarized in Table 4a,b. As shown in Figure 6, the absorption exhibits a major electronic absorption band in the visible region. For all of the examined dyes, the highest absorption occurs between 300 and 500 nm. The maxima of the dyes presenting the phenyl give a very important LHE compared to the dyes bridged by the triazine. To maximize the photocurrent response, the LHE of the sensitizer should be as high as possible; this is the case with our compounds with phenyl linkers. The above results confirm and recommend that the structure of these dyes is the best model for a dye-sensitized solar cell system.

Furthermore, the computation in the present manuscript was verified when compared with the optical absorption of bi-anchored sensitizer dyes reported in the literature. As shown in Table 4, the dye-based imidazole exhibits optical absorption from 300–600 nm [55]. In addition, the organic bi-anchored dyes containing triphenylamine/phenothiazine donors, 2-cyanoacrylic acid acceptors, and symmetric double D-π-A with arylamines as donors present almost the same absorption bands in the region (300–550 nm) [56,57,58].

#### 3.3.3. Dyes@TiO_2_ Cluster

The crucial component of DSSC devices is the sensitizer. In order to capture incident photons, it must have strong and widespread optical absorption. In order to decrease charge recombination, the charge transfer must also be unidirectional. As a result, the sensitizers need to be well-conjugated and coplanar. The chelated bidentate binding modes have been kept here among the possible binding modes of the dyes with the (TiO_2_)_9_ cluster (see Figure 5). Numerous dyes with a carboxyl anchor group have been determined to be stable for this bidentate bridging mode [59,60].

**Figure 7:**
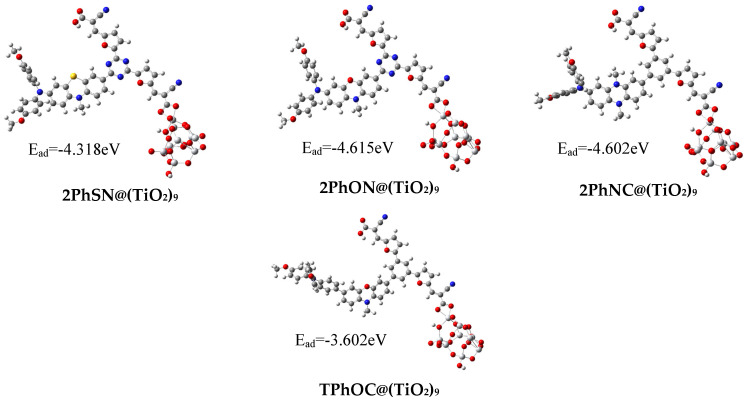
Structure of 2PhSN@(TiO_2_)_9_, 2PhON@(TiO_2_)_9_, 2PhNC@(TiO_2_)_9_, and TPhOC@(TiO_2_)_9_ **complexes**.

Figure 7 shows the structures of dyes@TiO_2_ optimized complexes. Bond lengths and torsional angles for these compounds reveal that they mostly remained unaltered during complexation with (TiO_2_)_9_. The length of every dye connection has been reduced from its free form to its complex form.

The results from the counterpoise correction method, taking into account the basis set superposition errors, are given in Figure 7. The complexation energies of the dyes (Dye@TiO_2_) resulting from this method are very low in absolute value. These energy values have an average of −4.248 eV. Thus, BSSE used in this study confirm its importance in calculating the complexation energy as previously reported [61,62].

**Figure 8:**
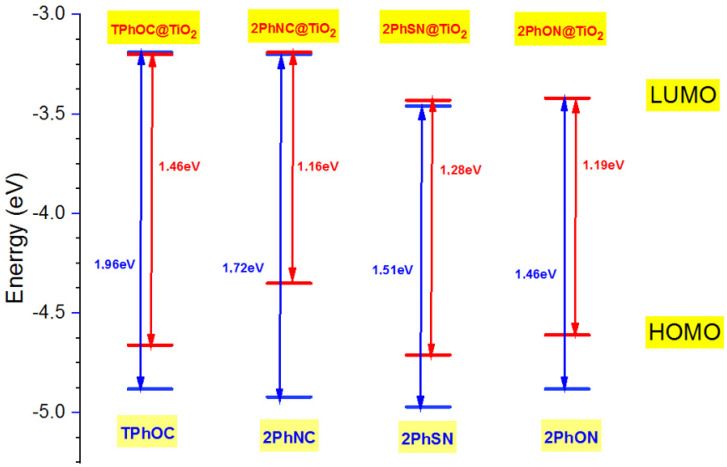
Comparative molecular orbital energies of the isolated and TiO_2_-bound studied dyes. The blue and red line represents, respectively, the position of the edge of the conduction and valence band for the isolated and complex dyes (Dye@TiO_2_).

It is observed that the HOMO orbital energies exhibit a change from the free dyes in terms of stabilization. The behavior of the molecular orbital energies of the dyes isolated and bound to TiO_2_ are shown in Figure 8. The energy destabilization is due to the interaction of the dye with the positive Ti(IV) surface ions and the transfer of electrons from the excited-state dye’s LUMO to the CB of the TiO_2_ cluster. It is noted that the LUMO orbital energies of the free dye are almost on the same level as those of the dyes calculated for the dye-TiO_2_ sensitizer, and the destabilization of HOMO upon interaction with the TiO_2_ cluster and electron density decreases the HOMO-LUMO energy gaps for adsorbed systems compared to free dyes.

The values of the gap energies of the dyes decrease after complexation with TiO_2_. It decreases in the following order: 2PhNC@TiO_2_ <2PhSN@TiO_2_ <2PhON@TiO_2_ <TPhOC@TiO_2_. All the complexed dyes show a band lower than the conduction band of TiO_2_, whereas the compound 2PhSN@TiO_2_ shows a near band of the iodine, which can present a regeneration of the charges compared to the other compounds. The compound 2PhSN@TiO_2_ has electronic properties that make it a better colorant.

## 4. Conclusions

The DFT and TD-DFT calculations have been performed for the newly designed dyes to analyze and understand organic dyes’ electronic structure, absorption, and transport properties. Changing donor (D, D’) and π-linkers in the dyes indicate different architectures. The dyes containing triazine π-linker are planar. However, the dyes containing phenyl π-linker are not planar. These results can modify the level of frontier orbitals. The triazine stabilizes the LUMO orbitals while the phenyl destabilizes them. On the other hand, the HOMO orbitals are stabilized for TPhOC, 2PhNC, 2PhSN, and 2PhOC, and allowed regeneration of the electron, compared with other dyes destabilized and do not have the regeneration. For the absorption, the maximum is located between 300–500 nm for the studied dyes. The absorption maxima of the dyes presenting the phenyl give a very important LHE compared to the dyes bridged by the triazine. The adsorption energy increases by modifying the π-linker. The values of the gap energies of the dyes decrease after complexation with TiO_2_. It decreases in the following order: 2PhNC@TiO_2_ <2PhSN@TiO_2_ <2PhON@TiO_2_ <TPhOC@TiO_2_. Using the counterpoise method when calculating complexation energies can express significant values in absolute terms, which is why it is always essential to consider this method when calculating complexation energies.

## Figures and Tables

**Table 1:** Values of dihedral angle (θ (°)) and bond length (Å) obtained by B3LYP/6-31G(d,p).

Dyes	d_1_(Å)	d_2_(Å)	θ (°)	Dyes	d_1_(Å)	d_2_(Å)	θ (°)
2PhNC	1.415	1.481	36.66	TPhNC	1.481	1.482	37.37
2PhOC	1.411	1.481	35.30	TPhOC	1.481	1.481	36.39
2PhSC	1.410	1.482	34.30	TPhSC	1.480	1.482	35.97
2PhNN	1.414	1.460	0.76	TPhNN	1.480	1.460	0.08
2PhON	1.407	1.462	0.04	TPhON	1.481	1.462	0.05
2PhSN	1.409	1.465	0.20	TPhSN	1.480	1.465	0.07

**Table 2:** **a**: Values of orbital molecular HOMO, LUMO, and gap energies calculated with B3LYP/6-31G(d,p) level for the studied donor-like triphenylamine-based dyes. **b:** Values of orbital molecular HOMO, LUMO, and gap energies calculated with B3LYP/6-31G(d,p) level for the studied donor-like diphenylamine-based dyes.

(a)				
Dyes		$E_{LUMO}$ (eV)	$E_{HOMO}$ (eV)	$E_{gap}$ (eV)
TPhNC	X = S.O.N Y = C	−3.20	−4.76	1.56
TPhOC		−3.19	−4.88	1.69
TPhSC		−3.12	−4.72	1.60
TPhNN	X = S.O.N Y = N	−3.33	−4.61	1.28
TPhON		−3.35	−4.67	1.32
TPhSN		−3.38	−4.72	1.34
(b)				
Dyes		$E_{LUMO}$ (eV)	$E_{HOMO}$ (eV)	$E_{gap}$ (eV)
2PhNC	X = S.O.N Y = C	−3.20	−4.92	1.72
2PhOC		−3.19	−4.80	1.61
2PhSC		−3.20	−4.64	1.44
2PhNN	X = S.O.N Y = N	−3.42	−4.71	1.29
2PhON		−3.42	−4.88	1.46
2PhSN		−3.46	−4.97	1.51

**Table 3:** **a:** The calculated redox potential of the ground state stable ($E_{ox}^{dye}$*in eV),* oxidation potential of the dye ($E_{ox}^{dye*}$ *in eV), absorption energy (*$E_{00}$  *in eV),* free energy change for electron injection ($\Delta G^{inj}\;in\;eV)$*, regeneration energy (*$\Delta G^{reg}$ *in eV), and recombination energy (*$\Delta G^{rec}$ *in eV) for dye-based triphenylamine.* **b**: The calculated redox potential of the ground state stable ($E_{ox}^{dye}$ *in eV),* oxidation potential of the dye ($E_{ox}^{dye*}$ *in eV), absorption energy (*$E_{00}$ *in eV*), free energy change for electron injection ($\Delta G^{inj}\;in\;eV)$*, regeneration energy (*$\Delta G^{reg}$ *in eV), and recombination energy (*$\Delta G^{rec}$ in eV) for dye-based diphenylamine.

**(a)**						
**Dyes**	$E_{ox}^{dye}$	$E_{00}$	$E_{ox}^{\mathrm{dye}*}$	$\Delta G^{\mathrm{inj}}$	$\Delta G^{\mathrm{reg}}$	$\Delta G^{\mathrm{rec}}$
TPhNC	4.76	3.22	1.54	−2.46	0.04	−0.76
TPhOC	4.88	3.23	1.65	−2.35	−0.08	−0.88
TPhSC	4.72	3.21	1.51	−2.49	0.08	−0.72
TPhNN	4.61	2.2	2.41	−1.59	0.19	−0.61
TPhON	4.67	2.86	1.81	−2.19	0.13	−0.67
TPhSN	4.72	3.04	1.68	−2.32	0.08	−0.72
**(b)**						
**Dyes**	$E_{\mathrm{ox}}^{\mathrm{dye}}$	$E_{00}$	$E_{\mathrm{ox}}^{\mathrm{dye}*}$	$\Delta G^{\mathrm{inj}}$	$\Delta G^{\mathrm{reg}}$	$\Delta G^{\mathrm{rec}}$
2PhNC	4.92	3.21	1.71	−2.29	−0.12	−0.92
2PhOC	4.8	3.23	1.57	−2.43	0.00	−0.80
2PhSC	4.64	3.23	1.41	−2.59	0.16	−0.64
2PhNN	4.71	2.43	2.28	−1.72	0.09	−0.71
2PhON	4.88	3.18	1.70	−2.30	−0.08	−0.88
2PhSN	4.97	3.33	1.64	−2.36	−0.17	−0.97

**Table 4:** **a:** Calculated maximum absorption wavelengths ($\lambda_{max}$/nm), vertical excitation energy ($E_{ex}$), oscillator strengths (OS), and major contribution of the dye-based triphenylamine in dichloromethane solution under TD-BHandH/6-31G(d,p) level. **b**: Calculated maximum absorption wavelengths ($\lambda_{max}$/nm), vertical excitation energy ($E_{ex}$), oscillator strengths (OS), and major contribution of the dye-based diphenylamine in dichloromethane solution under TD-BHandH/6-31G (d,p) level.

(**a**)						
Dyes	$E_{ex}\;(\mathrm{eV})$	$\lambda_{max}\left(\mathrm{nm}\right)$	$\lambda_{exp}\left(\mathrm{nm}\right)$ for Similar Dyes	OS	**LHE**	Major Contribution
TPhNC	2.58	480.12	398/ 400/ 394/ 418/ 388 [55]	0.0188	0.042	H-1→L(17.2), H→L(67.6)
	2.85	434.52		0.0114	0.025	H-1→L+1(15.1), H→+1(68.3)
	3.22	384.46		2.237	**0.994**	H-4→L(25.1), H-2→L+1(47.78), H-1→L(14.9)
TPhOC	2.89	428.14		0.0784	0.165	H-1→L(29.1), H→L(62.6)
	3.15	392.88		0.0646	0.138	H-1→L+1(23.2), H→L+1(64.1)
	3.23	383.07		2.1484	**0.992**	H-1→L(47.4), H→L(11.1), H→L+1(11.6)
TPhSC	2.93	423.06		0.0567	0.122	H-1→L(27.1), H→L(62.7)
	3.14	393.91		0.1541	0.298	H-1→L+1(22.2), H→L+1(61.9)
	3.21	385.61		1.4887	**0.967**	H-2→L+1(36.3), H→L(25.1), H→L+1(21.8)
TPhNN	2.25	550.1		0.0037	0.008	H→L(65.9)
	2.52	490.78		0.3687	**0.572**	H-1→L+1(12.7), H→L+1(59.1)
	2.96	417.98		0.0102	0.023	H-1→L(62.7), H→L+1(21.9)
TPhON	2.62	473.13		0.0001	0.001	H→L(62.8)
	2.86	432.7		0.5146	**0.694**	H→L+1(53.7), H→L+2(21.3)
	3.11	398.29		0.0191	0.043	H-1→L+1(60.1), H→L+1(30.4)
TPhSN	2.87	445.43		0.0002	0.001	H→L(64.8)
	3.04	407.27		0.3538	**0.557**	H→L+1(56.5), H→L+2(15.8)
	3.2	386.88		0.0084	0.019	H-1→L (62.6), H→L(26.4)
(**b**)						
Dyes	$E_{\mathrm{ex}}\;(\mathrm{eV})$	$\lambda_{\max}\left(\mathrm{nm}\right)$	$\lambda_{exp}\left(\mathrm{nm}\right)$ for Similar d=Dyes	OS	**LHE**	Major Contribution
2PhNC	2.46	502.88	370/ 427/ 444 [56] 320, 403/322, 422/382, 439 [57]	0.012	0.027	H-1→L(11.9), H→L(68.9)
	2.73	454.14		0.0091	0.02	H→L+1(69.3)
	3.21	385.14		2.2556	**0.994**	H-2→L+1(47.5), H-1→H(15.4)
2PhOC	2.71	456.5		0.0312	0.069	H-1→L(13.7), H→L(68.88)
	2.97	416.95		0.0143	0.032	H-1→L+1(10.93), H→L-1(69.9)
	3.22	383.95		2.2926	**0.994**	H-2→L+1(48.5), H-1→H(15.14)
2PhSC	2.42	511.41		0.004	0.009	H-1→L(13.6), H→L(68,3)
	2.68	461.07		0.3951	0.597	H→L+1(62.2), H→L+2(24.2)
	3.18	389.46		0.4317	**0.629**	H-1→L+1(20.3), H→L+2(52.5), H→L+5(15.4)
2PhNN	2.15	575.16		0.0029	0.006	H→L(68.3)
	2.43	508.68		0.3205	**0.521**	H→L+1(63.1)
	2.96	418.84		0.2632	0.454	H-1→L+1(29.1), H→L+2(56.3), H→L+5(15.9)
2PhON	2.42	511.41		0.004	0.009	H-1→L(13.6), H→L(68.3)
	2.68	461.07		0.03951	0.087	H-1→L+2(62.2), H→L+2(24.2)
	3.18	389.46		0.4317	**0.629**	H-1→L+1(20.3), H→L+2(52.5), H→L+5(15.4)
2TPhSN	2.62	472.86		0.0004	0.001	H-1→L(14.7), H→L(68.2)
	2.88	429.64		0.2779	0.472	H-1→L+1(15.4), H→L+1(62.5)
	3.33	371.23		0.3901	**0.592**	H-1→L+2(21.3), H→L (25.5), H→L+1(38.9)

## Data Availability

All data are presented in the article and Supplementary Material.

## Supplementary Materials

The following supporting information can be downloaded at: https://www.mdpi.com/article/10.3390/ma16041611/s1, Figure S1: Figure S2: the photovoltaic parameters.
