# Supplementary material for: The Impact of TPA Auxiliary Donor and the π-Linkers on the Performance of Newly Designed Dye-Sensitized Solar Cells: Computational Investigation
Source: Materials (Basel). 2023 Feb 15;16(4):1611. doi: 10.3390/ma16041611 (PMC9965092; doi:10.3390/ma16041611)
Supplement: Supplementary file 1 [file materials-16-01611-s001.zip › materials-2056102-SI.pdf]

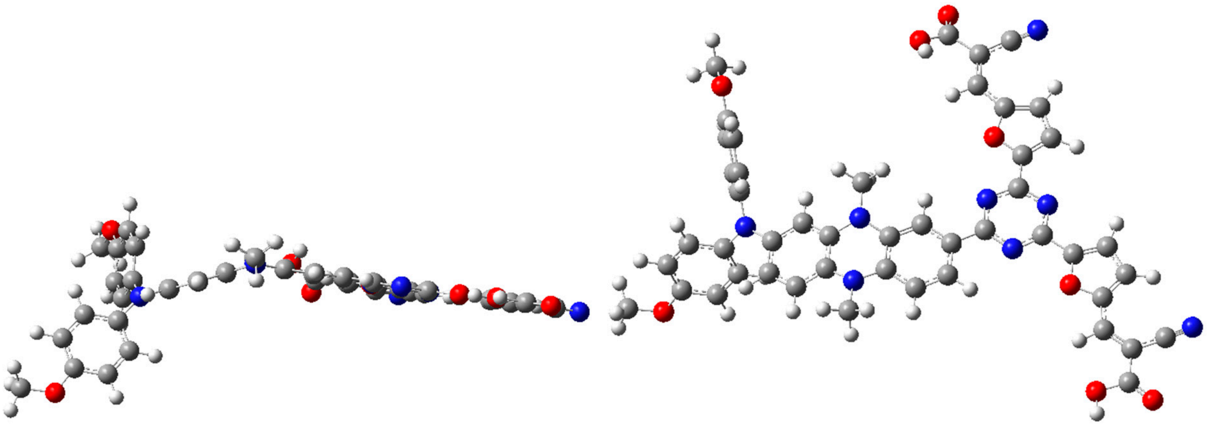

2PhNN

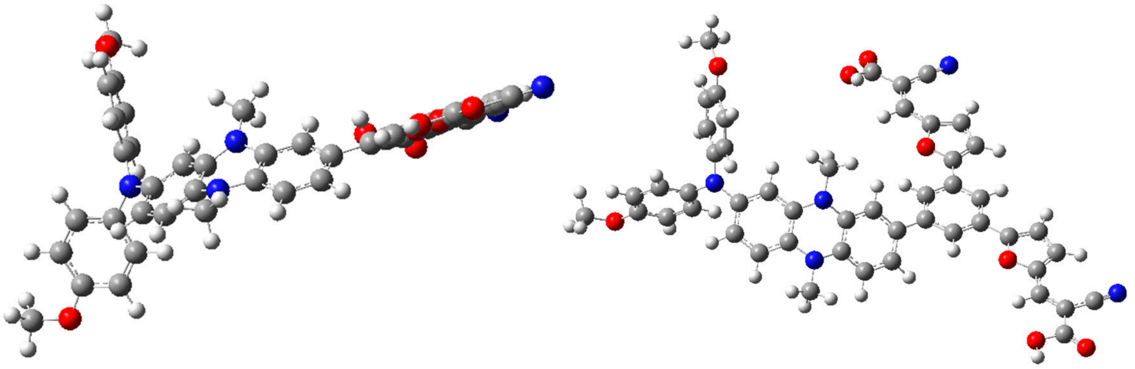

2PhNC

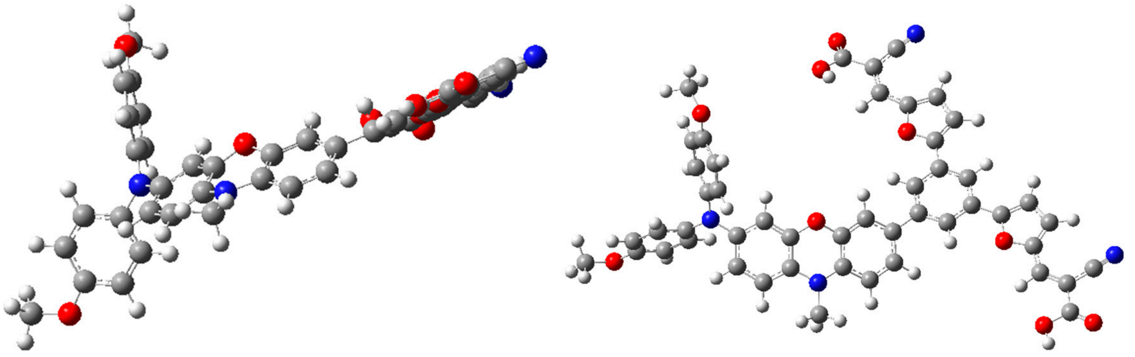

2PhOC

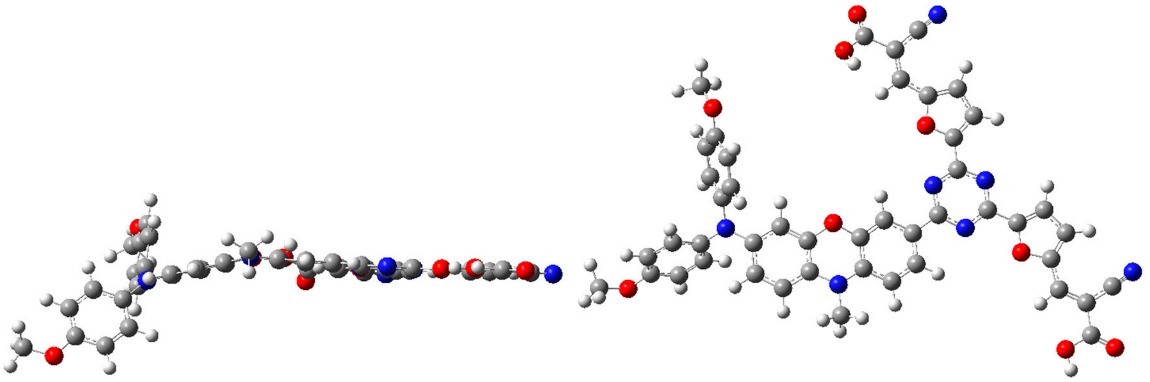

2PhON

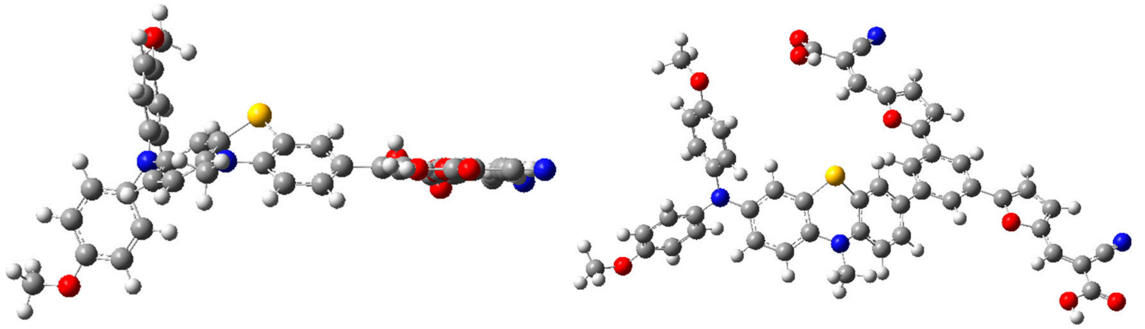

2PhSC

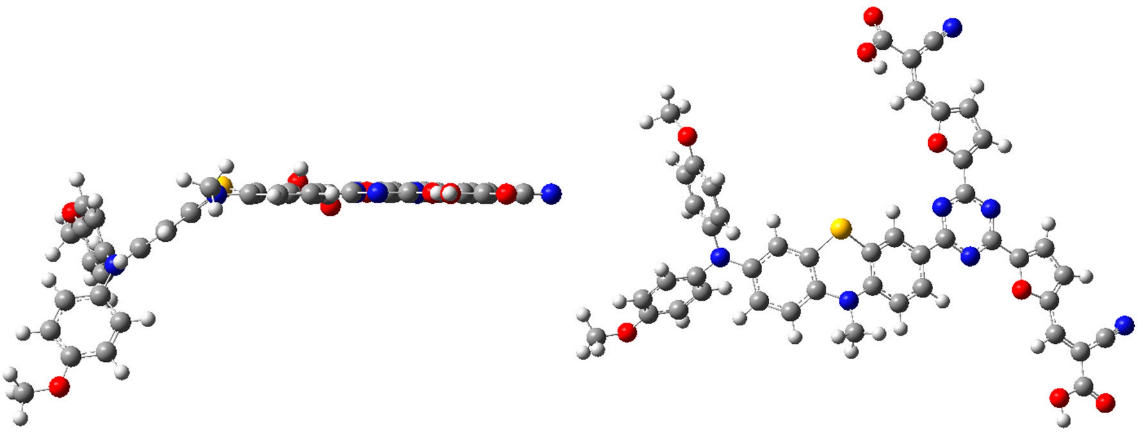

2PhSN

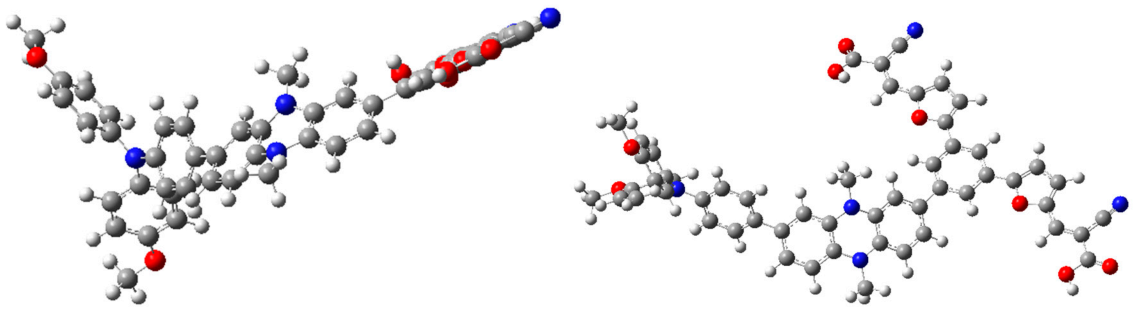

TPhNC

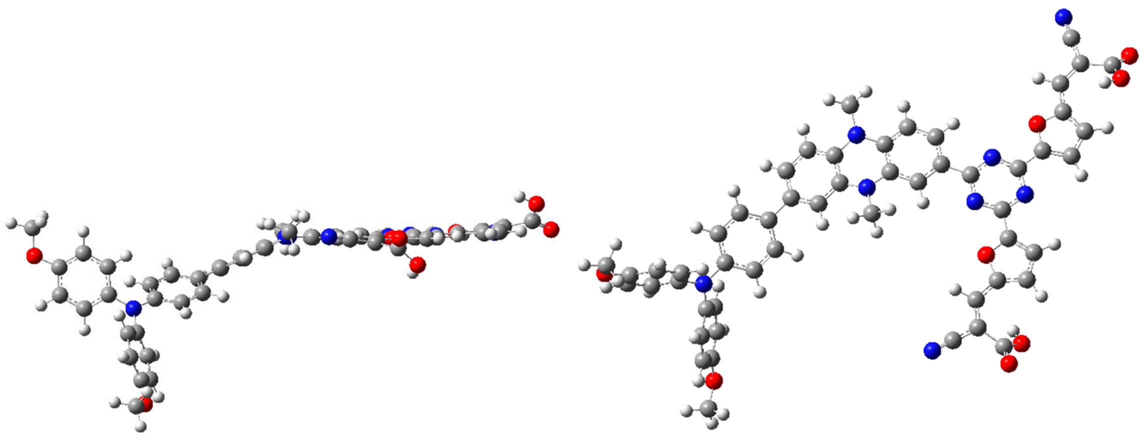

TPhNN

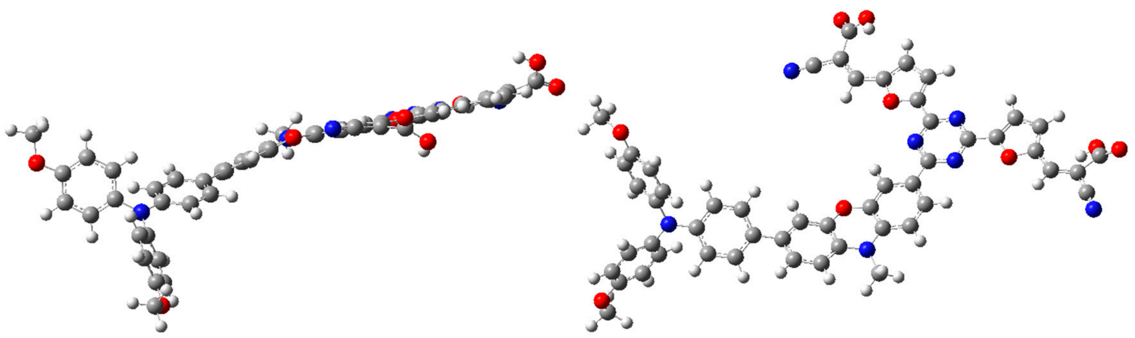

TPhON

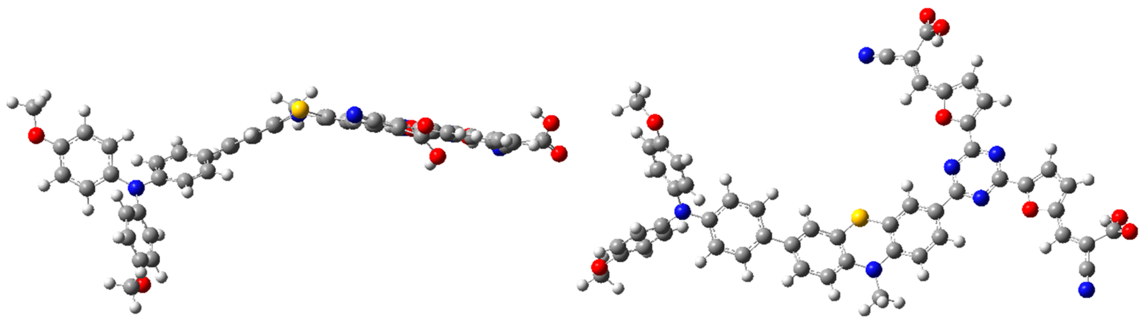

TPhSN

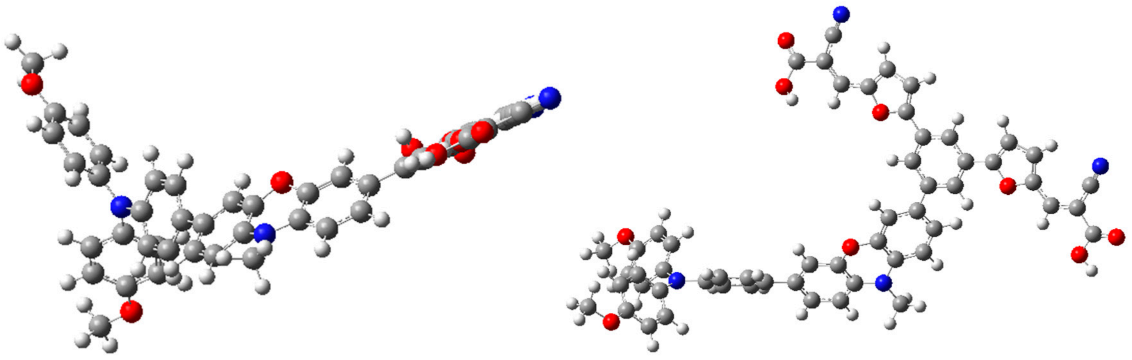

TPhOC

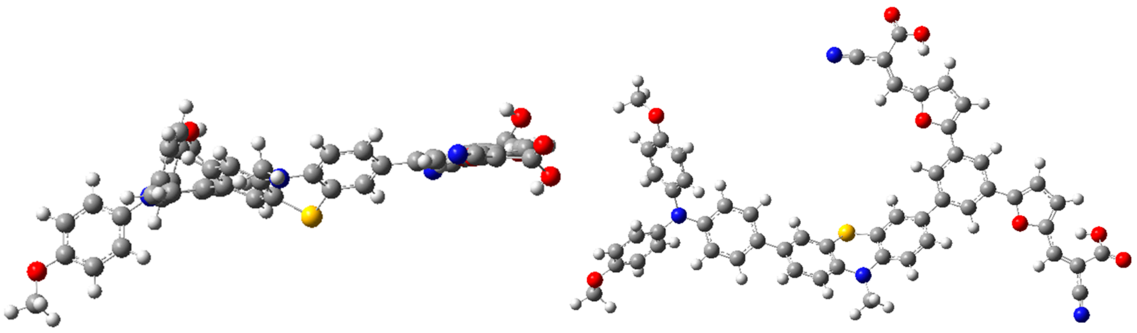

TPhSC

Figure S1: Optimized structure of the studied dyes.

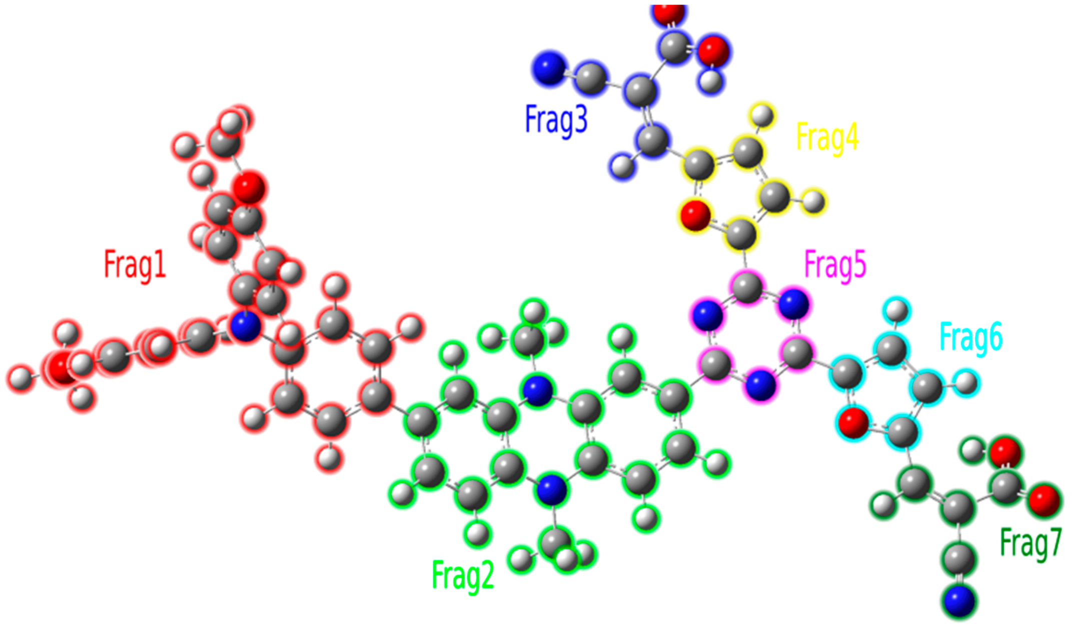

Figure S2: The structure of dyes with different fragments.

Theoretical background

Generally, the efficiency PCE of DSSCs can be calculated from the V_{oc}, J_{sc}, fill factor (FF) and the incident solar power on the cell (P_{in}) and the maximum power (P_{max}). The calculated efficiency can be expressed as follows [1,2]:

$$\boldsymbol{PCE(\eta\%) = \frac{P_{max}}{P_{in}} = \frac{V_{oc}J_{sc}}{P_{in}} FF.100\%} \quad (1)$$

The fill factor (FF) is defined as the ratio of the maximum power that the battery can yield to the theoretical maximum output power of the product of the J_{sc} and V_{oc}.

The J_{sc} is a key parameter for the DSSCs devices, which can be calculated with the following equation (2):

$$\boldsymbol{J_{sc} = q \int_0^{\infty} LHE(\lambda)\eta_{inj}\eta_{coll} \times I_s(\lambda)d\lambda} \quad (2)$$

Where *LHE*(λ) symbolizes the Light-Harvesting Efficiency and η_{inj} is the electron injection efficiency, η_{coll} is the charge collection efficiency, and $I_s(\lambda)$ is the corresponding photon flux of the solar radiation spectrum at a fixed wavelength.

Where $LHE(\lambda)$ symbolizes the light-harvesting efficiency, and ηinj is the electron injection efficiency, ηcoll is the charge collection efficiency, and $I_S(\lambda)$ is the corresponding photon flux of the solar radiation spectrum at a fixed wavelength.

The light harvesting efficiency is determined by:

$LHE(\lambda) = 1 - 10^{-f}$

Where f is the oscillator strength corresponding to the maximum absorption wavelength. The understanding of the molecular electronic properties in the excited state of the studied dyes is crucial to predict the solar cells properties of DSSCs. The efficiency of photovoltaic cells depends on the rate of electron injection and electron regeneration. The rate of electron injection (ΔG^{inj}) is defined as [3]:

$$\mathbf{\Delta G_{inj} = E^{*}_{dye} - E_{CB}} \quad (3)$$

E^{*}_{dye} : The oxidation of the excited dye and E_{CB} is the conduction band of the TiO_2.

$$\mathbf{E^{*}_{dye} = \boldsymbol{E_{dye}} - \boldsymbol{E_{0-0}}} \quad (4)$$

E_{dye} : The redox potential of the ground state of the dye and E_{0-0} is the vertical transition energy associated with the λ_{max}. The rate of electron regeneration ΔG_{reg} is defined as follows:

$$\boldsymbol{\Delta}\mathbf{G_{reg}} = \boldsymbol{E}_{(\mathrm{I}^-/\mathrm{I}_3^-)} - \mathbf{E_{dye}} \quad (5)$$

$E_{(\mathrm{I}^-/\mathrm{I}_3^-)}$ is the redox potential energy of (I^- / I_3^-). When the rate of electron recombination ΔG_{rec} is defined as: $\Delta G_{\mathrm{rec}} = E_{\mathrm{CB}} - \mathrm{E_{dye}}$

The processes of electrons injection, dye regeneration, and electrons transfer play a significant role in the overall power conversion efficiency. Large electron injection result in a high power conversion efficiency. The values of E_{OX}^{dye}, E_{0-0}, E_{OX}^{dye*}, ΔG^{inject}, $|V_{RP}|$, ΔG^{reg} of all designed dyes calculated at a B3LYP/6-31G(d,p) level, and their values are listed in Table 3a.

References

[1] G.P. Smestad, M. Grätzel, Demonstrating electron transfer and nanotechnology: A natural dye-sensitized nanocrystalline energy converter, J. Chem. Educ. 75 (1998) 752–756. https://doi.org/10.1021/ed075p752.

[2] Z. Zhao, G. Yu, Q. Chang, X. Liu, Y. Liu, L. Wang, Z. Liu, Z. Bian, W. Liu, C. Huang, Carbazolylphosphines and carbazolylphosphine oxides: Facilely synthesized host materials with tunable mobilities and high triplet energy levels for blue phosphorescent OLEDs, J. Mater. Chem. C. 5 (2017) 7344–7351. https://doi.org/10.1039/c7tc01594a.

[3] R. Katoh, A. Furube, Electron injection efficiency in dye-sensitized solar cells, J. Photochem. Photobiol. C Photochem. Rev. 20 (2014) 1–16. https://doi.org/10.1016/j.jphotochemrev.2014.02.001.
